# Involvement of GJA1 and Gap Junctional Intercellular Communication between Cumulus Cells and Oocytes from Women with PCOS

**DOI:** 10.1155/2020/5403904

**Published:** 2020-02-28

**Authors:** Qiwei Liu, Liang Kong, Junhui Zhang, Qian Xu, Jingxue Wang, Zhigang Xue, Jinjuan Wang

**Affiliations:** ^1^Department of Gynecological Minimal Invasive Center, Beijing Obstetrics and Gynecology Hospital, Capital Medical University, Beijing 100010, China; ^2^Anhui Province Key Laboratory of Reproductive Health and Genetics, Anhui Medical University, Hefei, 230032 Anhui, China; ^3^Translational Center for Stem Cell Research, Tongji Hospital, Department of Regenerative Medicine, Tongji University School of Medicine, Shanghai, China

## Abstract

Polycystic ovary syndrome (PCOS) is a common female endocrine system disease that affects 17.8% of women of reproductive age and leads to infertility, obesity, glucose metabolic disorders, cardiovascular disease, and body-mind problems. However, the etiology of PCOS remains unclear. Follicular growth is disrupted as a result of ovarian hyperandrogenism and distorted intraovarian paracrine signaling in women with PCOS. Microcommunication between oocytes and cumulus cells plays a critical role in folliculogenesis. Gap junction alpha 1 (GJA1) plays a crucial role in the developing follicles by forming communication channels between cumulus cells and oocytes, but this has not yet been reported in women with PCOS. Therefore, we aimed to study the role of GJA1 in the microcommunication between oocytes and cumulus cells in women with PCOS. In our study, cumulus cell-oocyte complexes (COCs) from women were isolated via ultrasound-guided vaginal puncture, and oocytes were selected from COCs and categorized based on 3 oocyte maturation stages. Then, RT-qPCR and immunofluorescence analysis were performed to detect both the gene expression and protein of GJA1 in oocytes from women with and without PCOS. There was no statistically significant difference in age and BMI (body mass index), but patients with PCOS had a higher ratio of basic LH/FSH (luteinizing hormone/follicle-stimulating hormone), androstenedione, and total ovarian volume. The qRT-PCR results showed higher gene expression of GJA1 in oocytes without PCOS at the germinal vesicle (GV) stage compared with that of oocytes from women with PCOS. Immunofluorescence analysis showed that the expression level of GJA1 in oocytes from women with PCOS was very weak compared with that of oocytes from women without PCOS. In conclusion, GJA1 may play a critical role in the development of oogenesis arrest in women with PCOS throughout the oogenesis processes, including oogenesis and oocyte maturation.

## 1. Introduction

Polycystic ovary syndrome (PCOS), first identified in 1935 by Stein and Leventhal, is a female endocrine disorder characterized by hyperandrogenism, ovulatory dysfunction, and polycystic ovarian morphology that affects 17.8% of women of reproductive age [1–3]. PCOS causes a series of major systemic complications, including infertility, obesity, glucose metabolic disorders, cardiovascular disease, and body-mind problems, which impair female health [4–6]. Among these complications, infertility is one of the most challenging problems for reproductive women, accounting for 80% of anovulatory infertility cases in women with PCOS [7]. The etiology of PCOS remains unclear. Follicular growth is disrupted as a result of ovarian hyperandrogenism and distorted intraovarian paracrine signaling in women with PCOS, which makes follicle arrest at secondary follicle stage, and the size of follicles stays in the range of 2-9 mm [8]. The follicular microenvironment plays a crucial role in folliculogenesis. During folliculogenesis, oocytes receive important signaling and nutrition only through communication with the cumulus cells around them. Through this communication, oocytes and cumulus cells complete folliculogenesis, oocyte maturation, and ovulation [9–12]. Therefore, microcommunication plays a critical role in oocyte maturation.

Gap junctional intercellular communication (GJIC) has been reported to participate in many normal physiological and pathological processes. Gap junctions (GJs) are essential channels linking adjacent cells and allow for the exchange of molecules, nutrients, and signaling molecules between cells [13, 14]. GJs encoded by connexins (Cxs) are a family of approximately 20 proteins that form gap junction channels that allow intercellular communication [15, 16]. GJA1, also called connexin 43 (Cx 43), was documented to play a crucial role in forming communication channels that couple developing follicles to cumulus cells, assisting in communication between cumulus cells and oocytes. Studies have reported that folliculogenesis was arrested when GJA1 was knocked out in the mouse ovary, and the follicles stayed in the primary stage and developed incompetent oocytes [17]. And inhibiting expression level of GJA1 in granulosa cells in rats can cause inhibition of progesterone production [18]. With GJA1, oocytes obtain nutrients, ions, and signals from somatic cells around them to support oocyte maturation, regulate pH, and maintain meiotic arrest [19, 20].

However, no studies have identified a role for GJA1 in oocytes and cumulus cells in follicles of women with PCOS. Our previous study showed that GJA1 was significantly differentially expressed between human oocytes with without PCOS [21]. In our study, we found that GJA1 expression was lower in PCOS oocytes at the GV stage than in healthy oocytes through single-cell RNA sequencing. To investigate the importance of GJA1 in the folliculogenesis arrest in PCOS, we further studied GJA1 in oocytes from women with PCOS.

## 2. Materials and Methods

### 2.1. Study Population

We recruited 16 women, including 8 women with PCOS and 8 without PCOS. All participants were undergoing assisted reproductive technology (ART) at Anhui First People's Hospital in Anhui, China, and were willing to donate oocytes. The study protocol was approved by the Research Ethics Committee of Anhui First People's Hospital (No. 2014008) and conducted in accordance with approved institutional guidelines. All participants gave written informed consent. PCOS patients were confirmed to have at least two of the three Rotterdam 2003 criteria for diagnosing PCOS: hyperandrogenism, oligoovulation and/or anovulation, and polycystic ovaries [22]. We excluded patients with Cushing's syndrome, congenital adrenal hyperplasia, and androgen-secreting tumors. Participant demographic and clinical characteristics, such as age, BMI, and LH, FSH, estradiol (E2), and progesterone levels, were recorded (Table 1).

### 2.2. Isolation of Oocytes

Ovarian stimulation for all participants was carried out according to the standard protocol. Cumulus cell-oocyte complexes (COCs) were isolated via ultrasound-guided vaginal puncture and washed in phosphate-buffered saline (PBS). Oocytes were selected from COCs and categorized based on the oocyte maturation stage: GV (oocyte maturation arrested in the prophase of meiosis), MI (metaphase I, first meiotic metaphase), and MII (metaphase II, second meiotic metaphase). Oocytes were prepared for use in RNA-seq experiments, and the remaining samples were prepared for quantitative reverse-transcriptase polymerase chain reaction (qRT-PCR) and immunofluorescence.

### 2.3. RNA Extraction and Real-Time PCR

RNA was extracted from single oocytes and converted into cDNA according to the Smart-seq2 protocol [23]. RNA was isolated from single oocytes using oligo-Dt30VN primers and dNTPs at 72°C and then reverse transcribed into cDNA. After the first-strand reaction, cDNA was amplified and purified twice by using AMPure XP beads and 80% ethyl alcohol. Significantly differentially expressed genes were confirmed by qRT-PCR (quantitative reverse-transcriptase polymerase chain reaction) (208054, QIAGEN, Germany).

The sequences of the primers for GJA1 and glyceraldehyde-3-phosphate dehydrogenase (GAPDH) used in this study are shown in Table 2. GAPDH was used to normalize the gene expression of GJA1. qRT-PCR was performed in a total reaction volume of 25 *μ*L, including 1 *μ*L cDNA (1 ng/*μ*L), 10 *μ*L 2x SYBR green PCR master mix, 0.1 *μ*L QN ROX reference dye, 1 *μ*L forward primer (10 mmol/L), 1 *μ*L reverse primer (10 mmol/L), and 6.9 *μ*L RNase-free water. The PCR initial activation was achieved by heating the samples to 95°C for 2 min, followed by a total of 40 cycles of denaturation at 95°C (5 s) and 60°C (30 s).

### 2.4. Immunofluorescence

Oocytes that were extracted from COCs were washed with PBS three times and then fixed for 10 min at room temperature in 4% paraformaldehyde. The oocytes were washed in 0.5% Triton-100 three times and treated with 0.5% Triton X-100 at room temperature for 40 min. Then, the oocytes were washed in 3% normal goat serum three times and blocked with 3% normal goat serum for 60 min. After that, the oocytes were washed once and then incubated with primary antibody (1 : 50 dilution, mouse anti-GJA1 monoclonal antibody) at 4°C overnight.

Then, the oocytes were washed with 3% normal goat serum three times. Subsequently, the oocytes were washed once and then incubated with goat anti-mouse immunoglobulin G fluorescein second antibody (1 : 100 dilution) for 1 h at room temperature. The oocytes were washed with 3% normal goat serum three times. After washing, the DNA in the cells was labeled with propidium iodide (PI) (1 : 2000 dilution) for 10 min. Finally, the oocytes were placed in a drop of 3% normal goat serum and observed under a fluorescence microscope.

### 2.5. Gene Expression Analysis and Statistics

Differential gene expression was considered significant if the adjusted *P* (Padj) < 0.05. Padj was calculated by using the Benjamini-Hochberg method to exclude false-positive results. All clinical parameters are expressed as the mean ± standard deviation depending on the distribution.

## 3. Results

### 3.1. Clinical and Biochemical Characteristics of Patients

The clinical characteristics of the 16 participants were analyzed and are shown in Table 1. There was no statistically significant difference in age or BMI, but patients with PCOS had increased LH (13.4 ± 1.3), ratio of basic LH/FSH (6.8 ± 0.6), androstenedione and total ovarian volume.

### 3.2. qRT-PCR Analysis

**Figure 1:**
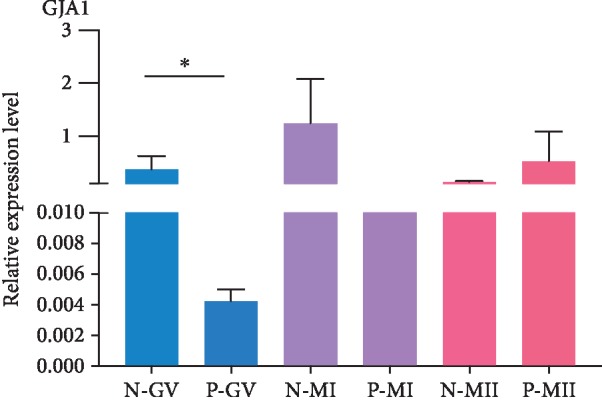
Differentially expressed genes in oocytes from PCOS compared with oocyte from non-PCOS by RT-PCR. N-GV: gene expression levels in oocyte at GV stage from normal women; P-GV: gene expression levels in oocyte at GV stage from PCOS women; N-MI: gene expression levels in oocyte at MI stage from normal women; P-MI: gene expression levels in oocyte at MI stage from PCOS women; N-MII: gene expression levels in oocyte at MII stage from normal women; P-MII: gene expression levels in oocyte at MII stage from PCOS women. ^∗^*P* value < 0.05.

We randomly selected 43 oocytes, which were divided into the GV, MI, and MII groups according to the oocyte maturation stage (Table 3). The qRT-PCR results showed higher gene expression of GJA1 in oocytes without PCOS at the GV stage compared with that of oocytes with PCOS, which corresponded to the results of single-cell RNA-seq in a previous article (Figure 1). However, there were no significant differences in gene expression at the MII stages between oocytes with or without PCOS.

### 3.3. Immunofluorescence Analysis

**Figure 2:**
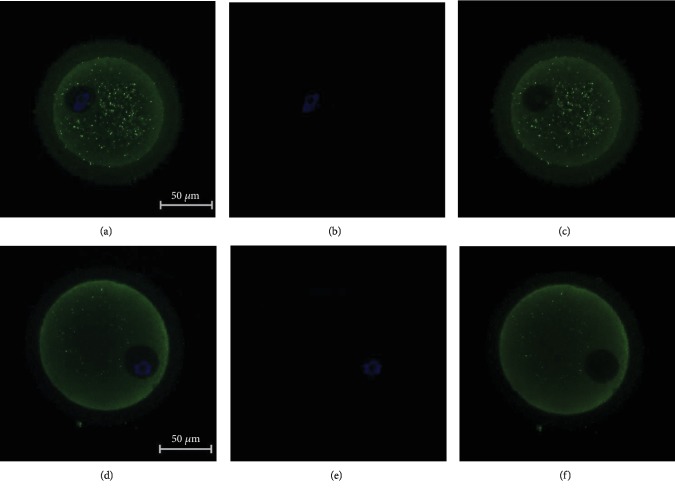
Immunofluorescence staining for detecting the expression pattern of GJA1 in human oocyte with and without PCOS at GV stage. (a–c) Oocyte from women without PCOS. (d–f) Oocyte from PCOS women. Oocytes were counterstained with the cytoskeleton (green) and nuclei (DAPI, blue). Bar = 50 *μ*m.

To detect the expression pattern of GJA1 in oocytes, immunofluorescence analysis was performed (Figure 2). The results showed a positive fluorescence signal for GJA1 in oocytes both with and without PCOS at stage GV. Moreover, the expression level of GJA1 in oocytes from women with PCOS was much weaker compared with that of oocytes from women without PCOS.

## 4. Discussion

In this study, we found that GJA1 was negatively expressed at both the gene and protein levels in oocytes with PCOS compared with non-PCOS oocytes, which was consistent with the outcomes of our previous study. These data suggest that GJA1 downregulation may be involved in folliculogenesis arrest in women with PCOS, which causes anovulatory infertility in these women. Androstenedione may play a role in the expression level of GJA1 both in rat polycystic ovary [18]. Our study also suggested that the gene expression of GJA1 was not different between oocytes from women with or without PCOS at the late stage of oogenesis after ART. This result indicates that ART may improve maturation capacity by regulating gap junctions.

Oocytes in follicles are tightly surrounded by cumulus cells and cannot directly obtain signals and nutrition from the microenvironment of the ovary. Therefore, cellular interaction between oocytes and somatic cells plays an indispensable role in oocyte growth and maturation and folliculogenesis [24–26]. One study showed that mice with disrupted GJs failed to undergo oocyte meiotic maturation and were infertile [17]. Gap junction intercellular interactions are maintained between somatic cells and oocytes during the early stage of oogenesis and are blocked by GJA1 phosphorylation when oocyte meiosis begins as the last stage [27, 28]. GJs provide nucleotides, amino acids, and energy substrates that the growing oocyte is unable to obtain itself from neighboring cells [29]. Several studies have suggested that deletion of Gja1 in granulose cells of mice prevents oocyte development [17, 30]. In addition, cumulus cells also help to maintain a stable intracellular pH in oocytes through gap junction channels, which is a fundamental homeostatic process for the survival and proliferation of oocytes [31]. Communication with granulosa cells also promotes chromatin structure remodeling during the final phase of bovine oocyte differentiation and acquisition of meiotic competence [32].

At the last stage of folliculogenesis, MAPK is stimulated by LH through the ERK-MAPK signaling pathway and promotes GJA1 phosphorylation [33]. With GJA1 phosphorylation, the gap junction is lost, which decreases the concentration of cGMP (cyclic guanosine monophosphate) in oocytes and promotes the process of meiosis [34–36]. cAMP (cyclic adenosine monophosphate), a second messenger, has been known to induce oocyte meiotic processes. cGMP inhibits the hydrolysis of cAMP in oocytes, and so a low concentration of cGMP induces the hydrolysis of cAMP, which promotes the meiotic process and oocyte maturation.

In conclusion, GJA1 may play a critical role in the development of oogenesis arrest in women with PCOS through all of the oogenesis processes, including oogenesis and oocyte maturation. Our study may provide a clue to determining the etiology of PCOS.

## Figures and Tables

**Table 1:** Clinical characteristics and outcome of patients with and without PCOS.

	Non-PCOS (*n* = 8)	PCOS (*n* = 8)	*P* value
Age (year)	26.4 ± 1.1	25.9 ± 0.8	0.7035
BMI (kg/m^2^)	23.6 ± 0.1	23.1 ± 1.7	0.7804
AFC	7.8 ± 0.5	>24.000	<0.001 (0.000)
FSH (mIU/mL)	7.4 ± 0.7	7.6 ± 0.3	0.7820
LH (mIU/mL)	5.9 ± 0.8	13.4 ± 1.3	<0.001 (0.000)
LH/FSH	3.5 ± 0.5	6.8 ± 0.6	<0.001 (0.000)
Insulin (IU)	12.4 ± 1.7	15.4 ± 4.4	0.8541
Testosterone (nmol/L)	0.9 ± 0.1	1.9 ± 0.2	<0.001 (0.000)
Androstenedione (nmol/L)	1.8 ± 0.1	2.3 ± 0.3	0.1611
SHBG (nmol/L)	54.2 ± 7.0	40.0 ± 4.8	0.1177

BMI: body mass index; FSH: follicle-stimulating hormone; LH: luteinizing hormone, AFC: antral follicle; SHBG: sex hormone-binding globulin.

**Table 2:** Primers used for qRT-PCR.

cDNA	Sense primer (5′-3′)	Antisense primer (5′-3′)
GJA1	CAATCTCTCATGTGCGCTTCT	GGCAACCTTGAGTTCTTCCTCT
GAPDH	ACCCGCCCTATCTCAACTACC	AGGACACCATAATGACAGCCT

GJA1: Gap junction alpha 1 protein; GAPDH: glyceraldehyde-3-phosphate dehydrogenase.

**Table 3:** Numbers of oocyte at different stages analyzed by qRT-RNA and immunofluorescence.

	GV	MI	MII	Immunofluorescence
Normal	6	6	4	5
PCOS	6	7	4	5

GV: germinal vesicle, oocyte maturation arrested in the prophase of meiosis; MI: metaphase I, first meiotic metaphase; MII: metaphase II, second meiotic metaphase.

## Data Availability

The data used to support the findings of this study are included within the article.
